# General practitioners’ challenges and strategies in dealing with Internet-related health anxieties—results of a qualitative study among primary care physicians in Germany

**DOI:** 10.1007/s10354-020-00777-8

**Published:** 2020-08-07

**Authors:** Julian Wangler, Michael Jansky

**Affiliations:** Centre for General and Geriatric Medicine, University Medical Centre Mainz, Am Pulverturm 13, 55131 Mainz, Germany

**Keywords:** Cyberchondria, Health anxiety, Health information, Doctor–patient relationship, Primary health care, General practitioner, Cyberchondrie, Gesundheitsangst, Gesundheitsinformation, Arzt-Patient-Verhältnis, Primärversorgung, Hausarzt

## Abstract

Time and again, it is discussed that in medical practices, the number of patients who develop health anxieties due to extensive health information searches on the Internet is increasing. The objective of this study is to explore and describe general practitioners’ experiences and attitudes towards cyberchondria patients as well as strategies to stabilize affected patients. Following a qualitative approach, oral personal semi-standardized interviews with general practitioners (*N* = 38) in Rhineland-Palatinate, Germany, were conducted in 2019. In the course of a content analysis, one can see that most interviewees see the emergence of Internet-related health anxieties as an increasing problem in everyday care. Affected patients not only show marked levels of doubt and nervousness as well as hypersensitivity to their own state of health, but also low confidence in the physician. In addition to compliance-related difficulties, the high need for advice and the demand for further diagnostics are regarded as major problems. Various approaches were identified by which general practitioners respond to unsettled patients (more consultation time, recommendation of reputable websites, information double-checking, expanded history questionnaire, additional psychosocial training).

**Electronic supplementary material:**

The online version of this article (10.1007/s10354-020-00777-8) contains supplementary material, which is available to authorized users.

## Abstract (de)

Immer wieder wird darüber diskutiert, dass in ärztlichen Praxen die Zahl jener Patienten steigt, die aufgrund ausufernder Online-Recherchen zu Gesundheits- und Krankheitsthemen Ängste um ihre Gesundheit entwickeln. Ziel der vorliegenden Studie ist es, die Erfahrungen und Einstellungen von Hausärzten im Hinblick auf Patienten mit „Cyberchondrie“ zu explorieren und Strategien zur Stabilisierung dieser Patientenklientel zu erfassen. Im Zuge eines qualitativen Ansatzes wurden mündlich-persönliche, halbstandardisierte Interviews mit Hausärzten (*n* = 38) in Rheinland-Pfalz im Jahr 2019 durchgeführt. Die Inhaltsanalyse zeigt, dass die meisten Probanden das Aufkommen von internetassoziierten Gesundheitsängsten als zunehmendes Problem in der alltäglichen Versorgung sehen. Betroffene Patienten zeigen nicht nur ein erhebliches Ausmaß an Zweifeln und Nervosität sowie eine Hypersensitivität in Bezug auf den eigenen Gesundheitszustand, sondern zugleich ein geringes Vertrauen in den Arzt. Zugleich werden Compliance-Probleme, das ausgeprägte Bedürfnis nach patientenseitiger Rücksprache bzw. Diskussion und die Forderung nach weitergehender Diagnostik als erhebliche Probleme benannt. Verschiedene Ansätze wurden identifiziert, mit denen die Hausärzte versuchen, auf gesundheitsängstliche Patienten einzugehen und diese zu stabilisieren (mehr Beratungszeit, Empfehlung seriöser Internetseiten, Nachprüfung von recherchierten Informationen, Erweiterung des Anamnesefragebogens, psychosoziale Weiterbildung).

## Introduction

Today, many people use the Internet to get various information about health and illness issues [1–4]. The spectrum ranges from search engines to large health portals or special forums where one can interact with other users or even (medical) experts [5–7]. This also has an impact on the physician–patient relationship: When it comes to health issues, the doctor is no longer the first point of contact, since a search engine is increasingly being called up first. Thus, the doctors’ interpretation and decision-making authority over medical questions competes with other sources that are consulted by the patient. Consequently, the physician–patient relationship moves from autonomy towards consumerism [1, 3].

Basically, searching for health information online can be useful and helpful, for example by providing a better understanding of a medical diagnosis or therapy. Studies show that patients who do Internet research for preparation and follow-up in addition to medical consultations are better informed and are more health conscious. Moreover, health information can unfold positive effects on patients’ compliance or adherence [8–10]. However, under certain circumstances, extensive research on the Internet can reinforce or trigger health anxieties that can become permanently entrenched in the long run.

One example is the divergence between statements or suggestions for therapy from the doctor and recommendations on the Internet, which can lead patients to a loss of orientation and trust [11, 12]. By “Googling” complaints, symptoms, diagnoses, and therapies in an autonomous and increasingly excessive way, there is a risk of referring erroneous information from dubious websites or of drawing wrong conclusions from what has been read. Accordingly, restlessness, nervousness, and a state of fear can be the result of an intensified health-related online search.

In the worst case, such behavior can lead to cyberchondria [3, 13]. Patients with cyberchondria and patients of general hypochondriasis are often convinced to have disorders “with common or ambiguous symptoms” [14, 15]. Consequently, cyberchondria can be understood as an anxiety disorder or extreme attention to one’s own state of health triggered by unrestrained investigations on the Internet [16–18].

The psychological mechanism that is effective in the case of cyberchondria has hardly been studied so far. Based on the intention to exclude or to narrow down possible illnesses, the patient takes the clarification with regard to his/her health condition into his/her own hands by increasingly consulting the Internet [2, 4]. Thereby, the patient intends to better inform physicians about complaints and symptoms and to be able to initiate a possible therapy more quickly and purposefully. White and Horvitz assume that patients’ Internet searches are being used as a quasi-diagnostic method, using factors such as order, prominence, and informational content to draw conclusions about the quality and the representativity of the results [14, 19]. However, this behavior can easily result in misinterpreting nonspecific symptoms as a serious disease.

As the increasingly compulsive search on the Internet without medical consultation progresses, panic states and even delusions can be triggered and become entrenched [20, 21]. The patient comes to a tipping point, from which he/she acts more and more autonomously. Along with this, the patient gives the information he/she seeks great credibility and lacks confidence in doctors and medical advice [9, 22]. As a result, physicians may be expected to confirm self-diagnoses and to implement certain diagnostic or therapeutic measures, regardless of the plausibility of the patient’s assumptions and conclusions towards his/her state of health [6, 13, 23]. In extreme cases, patients may believe to be affected by fatal diseases for which there are no indications from a medical point of view [24, 25].

Although the scientific preoccupation with this topic is only at the beginning, it seems obvious that, at the individual level, previous experience (for example, chronic illnesses or illness experiences within the family environment) and mental and emotional personality predispositions do play a decisive role [7, 12, 26]. According to Eichenberg [27, 28], complex conditional structures are to be assumed, such as a moderating effect of health anxiety on the interrelation between Internet searching and health-related behavior [29, 30].

Only a few empirical studies have yet been presented that deal with the consequences of Internet-related health anxieties. International research indicates a correlation between the intensity of patients’ online searches and the use of medical appointments, diagnostic procedures, and health services [14, 23, 25, 31, 32]. However, certain studies show that intensive consultation of health information on the Internet may result in a reduction or even a termination of outpatient visits in the long-term [6].

For German-speaking countries, a first study on the relationship between health-associated Internet searching and hypochondria was conducted in 2013 [27]. Among the 471 respondents from over 180 online forums, 15% are classified as hypochondriacal. This group is more likely to search the Internet for complaints and symptoms. They also assess the quality of online health services as much higher and show a wider tendency to self-medicate. Depending on the severity of a hypochondriacal disorder, it can have a significant impact on things such as work ability, coping with everyday life, self-esteem, or resilience [28, 33–38].

To date, there is a lack of studies that examine the experiences that physicians have had with Internet-related health anxieties and how they respond to patients who are nervous or anxious as a result of intensive online searches [39]. A survey of 800 physicians from various disciplines has shown that 78% often or occasionally find the effects of (excessive) online research of patients in everyday care to be counterproductive and stressful [40]. An American study by Murray et al. [30] showed that physicians confronted with cyberchondria patients often feel limited in their ability to effectively act on the patient [1, 41].

Furthermore, physicians express concerns about patients who self-diagnose on the basis of online health information, since in many cases this is accompanied by demonstrating an incomplete or distorted understanding of other diagnostic possibilities and medical likelihoods [42]. For example, by exaggerating one set of symptoms in support of a self-diagnosis, the patient can minimize or suppress contrary symptoms. This impairs rather than enhances the doctor’s ability to reach a correct diagnosis [19, 24, 30].

Primary care is particularly affected by Internet-related health concerns. As the first point of contact for all questions about health disorders within the German and other health care systems, the general practitioner (GP) plays a key role in dealing with anxious and hypochondriacal patients. Patients usually have a great deal of confidence in their GP, so that primary care has good access to de-escalating and stabilizing those affected by cyberchondria and, if necessary, preparing them for professional psychotherapeutic care [9].

A qualitative study showed that general practitioners in Great Britain experience considerable anxiety in response to patients bringing information from the Internet [42]. A recent survey of 844 German GPs [43] demonstrated that the doctors surveyed observe a significant increase in the number of patients who have developed anxiety as a result of their overflowing Internet searches. Two-thirds of the primary care physicians state that 15% or more of their own patients regularly confront them with the results of their online searches. From the respondents’ experience, the patients are often confused by their investigations and respond in a nervous and anxious way. Only a minority of the respondents perceive positive effects of the web research such as a better understanding of the doctor or a timelier appearance in the practice. However, there are no study results on primary care procedures and strategies in doctor–patient communication on the subject.

In order to gain in-depth insights into general practitioners’ perceptions, experiences, and attitudes towards patients with Internet-related health anxieties/cyberchondria, this exploratory study addresses the following questions:

- How do GPs perceive patients who regularly and/or intensively search for information on health and illness on the Internet? What impact does the online health research have on the patients from the GPs’ experience?
- What experiences have GPs made in their everyday work with patients who develop fears on the basis of previous web searches? What do they consider as special challenges?
- What actions do they take to respond appropriately to unsettled patients or to avert the (further) emergence of cyberchondria?

The focus was on patients with marked anxiety disorders, where the general practitioner has reason to believe that the Internet search has a significant share of the health concerns or fears that arose. The aim is to make different approaches and strategies visible when it comes to stabilizing cyberchondria patients or preventing the emergence of health anxieties due to extensive online searches.

## Material and methods

### Interview content and data collection

Since little is known about how general practitioners deal with Internet-related health anxieties and which strategies they use to avert or counteract them, there is a need for a broader exploration of this issue. Consequently, a qualitative approach with semi-structured interviews appeared most appropriate. The interview guidelines were developed based on a literature review and findings from a quantitative preliminary study already mentioned [43]. In the course of the first interviews, the instrument was further specified.

The guidelines consist of 13 superordinate questions with several sub-questions and primarily focus on the following topics: Attitudes towards online-searching patients, (behavioral) observations and characteristics of online-searching patients and patients affected by cyberchondria, approaches and strategies for stabilizing cyberchondria patients (see Electronic supplementary material).

### Recruitment, study interviews, and participants

The recruitment took place by phone or email. A total of 46 general practitioners were contacted in the federal state of Rhineland-Palatinate, Germany, nine of whom refused an interview, mostly for reasons of time. The remaining 38 general practitioners who agreed to participate were interviewed by two researchers. All participants were sent the participant information sheet beforehand via email and this contained the aims of the study.

In order to gain a heterogeneous sample, various aspects were deliberately varied (practice type, urban or rural practice environment, age, gender, geographical distribution of the practices within Rhineland-Palatinate). Table 1 provides an overview of the participating samples.

**Table 1:** Sociodemographic characteristics of the sample

Sociodemographic characteristics (*N* = 38)	
Type of practice	50% (19) joint/group practice, 50% (19) solo practice
Practice location	45% (17) rural community/small town, 21% (8) medium-sized town, 34% (13) large city
Status	79% (30) practice owner, 21% (8) salaried physician
Age	Ø 53 years (minimum 36, maximum 71)
Sex	53% (20) male, 47% (18) female
Patients per quarter	26% < 1000, 33% 1000–1500, 41% > 1500

The semi-standardized interviews took place between April and July 2019 and were conducted orally and personally. Interviews were recorded using two digital voice recorders. The duration of the interviews ranged from 45 to 65 min.

### Data preparation and analysis

Interviews were transcribed verbatim. Each transcript was double-checked for inaccuracies. The theoretical saturation was reached so that further interviews were not required. The analysis of the transcripts was carried out with the use of MAXQDA software (VERBI Software, Berlin, Deutschland). The data were analyzed according to the method of qualitative content analysis based on Mayring [44].

In the course of the analysis, a category system was created which was repeatedly tested and modified as the analysis progressed. In this way it was possible to condense and systematize differences and similarities in the data in the form of arguments or problematic patterns. The created category system is based on the priorities set in the guidelines. As the paper focuses on primary care strategies for dealing with Internet-related health anxieties, the category system is presented below (see Table 3).

## Results

### Patients’ online search behavior in everyday practice

35 of the 38 interviewees believe that the proportion of patients searching the Internet for health- and disease-related information has risen significantly in recent years. Three quarters of the general practitioners assume that 15 to 20% of their own patients regularly conduct (extended) online searches. Some of these patients confront their doctors more frequently with the results of their investigations. According to the interviewees, the information that patients consult on the Internet almost always relates to clinical pictures or symptoms, therapies, diagnostic procedures, and (new) medicines. Other aspects, such as prevention, health services, healthy lifestyle, or additional medical care, only play a minor role.

There is a fixation on wanting to exclude diseases or to be as well informed as possible about manifestations, recognition, and treatment procedures. Some patients overdo their investigations at some point—and that can have negative effects (I‑2, female).

The interviewees state that in most cases, the online research is being used to follow up visits to the doctor in order to understand diagnoses better and to double-check the doctor’s actions or advice. However, according to the view of the general practitioners, it is becoming more and more common for patients to intensively search the Internet before visiting a doctor. Most interviewees are aware of patients who have decided to go to a medical consultation only because of their previous online research. Especially in such cases, it happens that patients come to their GPs with very specific beliefs, expectations, and wishes before the doctor can even get an idea of the patient’s state of health and any possible symptoms.

Sometimes these people literally overrun me. These patients are often very biased […]. And then, as a family doctor, you have the ‘joy’ of first understanding how the patient came to his/her ideas or the things that are being demanded from me. Sometimes you really have a hard time keeping up with such patients. These people have already drawn their conclusions, and that’s a huge problem (I-23, male).

### Occurrence and manifestations of Internet-related health anxieties/cyberchondria

The interviewees are familiar with the phenomenon that patients may be very unsettled or frightened due to previous online searches and, as a result, fear that they may have a serious illness, although from a medical point of view there is no such evidence. More than three quarters of the general practitioners interviewed indicate that such an unfounded fear of being seriously ill, based on the knowledge of Internet content, has often or at least occasionally attracted their attention and poses an increasing problem in primary care.

The interviewees identify different challenges in caring for cyberchondriacal patients. A high level of nervousness, anxiety, irrationality, and sometimes panic is most commonly mentioned. Corresponding to this, the tendency is described that patients constantly think about their health situation and relate much in their everyday life to it. In addition, the general practitioners feel controlled in their conclusions and their work by the patients who tend to double-check a diagnosis or a therapy via online search. From the experience of the interviewees, the patients often doubt the medical advice and ask critical questions. Because of this, those interviewed again and again find themselves in “uncomfortable justification situations” (I‑9, male).

Similarly challenging is the fact that cyberchondria patients show a pronounced willingness to engage in larger discussions and even conflictual disputes with the doctor. As a result, some interviewees state that cyberchondria patients have less overall trust in the doctor since they no longer know “what to believe” because of conflicting and/or false online information that leads to great confusion (I-31, female).

In addition to such compliance-related difficulties, the massive need for counseling of such patients is experienced as a key problem. Thus, they do not only make use of relatively vast health care capacities but cause delays in practice processes, which brings disadvantages for other patients.

Excessive Internet searches often do not contribute to a better understanding but increase the patients’ need for discussion and clarification because people are massively confused and unsettled. You can see that very well when you look at how many people come to my office hours (I-15, female).

The call for further diagnostics without comprehensible medical cause is considered as particularly problematic. Some of the general practitioners interviewed report a feeling that they have to act as “tools of long-established decisions of the patient” and, thus, feel restricted in their role and responsibility as physicians (I‑7, male).

A huge problem is that many patients no longer go along with an unbiased medical examination but more or less want to have their search results and their conclusions confirmed. There are complex examinations or certain referrals to medical specialists being demanded of me. […] If you as a doctor do not oppose that you will find yourself in a questionable role (I‑4, male).

Some of the interviewees are convinced that the excessive research on the Internet that can ultimately trigger or exacerbate a cyberchondria often goes along with damaging the physician’s authority.

Although the Internet offers a great deal of information, the more you consume it, the more you need a competent authority to classify these often contradictory information and relate them to the specific situation of the patient. At this point, however, the patient has often to some extent lost confidence in doctors and no longer knows whose advice he can build on. He/she enters a vicious circle (I-16, female).

One fifth of the sample states to have already experienced the termination of a patient-to-doctor basis due to the patient’s extensive online research. In these extreme cases, the behavior of patients is described as extremely unreasonable, disturbed, aggressive, or suspicious of the doctor. Patient care was stopped by the general practitioner or the patient because the latter was so strongly influenced by information from the Internet that further care was no longer possible.

I see a very big danger in the fact that the patient gets into a kind of tunnel through his/her constant search on the Internet and then, in the end, is no longer receptive to the doctor’s advice. Again and again, I experience those patients who constantly feel misunderstood and do doctor hopping (I-35, female).

Most interviewees find it difficult to describe “typical” (sociodemographic) characteristics of cyberchondria patients. In many cases, it is rather younger and better educated people with a high degree of sensitivity, sometimes people affected by psychosomatic disorders or with chronic illnesses in the past.

With regard to the question of which decisive cause is at the center of cyberchondria, the general practitioners are divided. One third of the sample considers only personality predispositions and a general tendency towards hypochondriacal behavior to be responsible for the emergence of such a psychiatric disorder. These interviewees assume that cyberchondria patients would most likely develop health fears even without searching on the Internet, only by other means.

The other general practitioners do not completely exclude a certain predisposition or family influence, but consider it possible that even individuals may progressively develop cyberchondria who in fact have no hypochondriacal tendencies. One reason for this is the “flood of information on the Internet which is difficult to control and can have unpredictable consequences” (I-15, female).

There is something specific about the medium Internet. […] The treacherous thing is that the patients do not perceive what happens to them through their research. This is a spiral, a gradual process of getting into it. The Internet is doing something with the people. In the end, they are convinced they have something terrible, they have physical pain and discomfort (I-26, male).

Table 2 gives an overview of the interviewees’ positions and experiences.

**Table 2:** Overview of main findings

Issue	Percentage share in the sample (*N* = 38)
Proportion of patients searching the Internet for health- and disease-related information has risen significantly in recent years	92% (35) has risen, 8% (3) no change
Percentage of patients regularly conducting (extended) online searches	76% (29): 15 to 20%; 10% (4): 10 to 15%; 13% (5): less than 10%
The information that patients consult on the Internet almost always relate to	92% (35) clinical pictures, 92% (35) symptoms, 79% (30) therapies, 74% (28) diagnostic procedures, 66% (25) (new) medicines (multiple entries were possible)
Online research is being used to follow up visits to the doctor in order to understand diagnoses better and to double-check the doctor’s actions or advice	74% (28) yes, 26% (10) no
Familiarity with the phenomenon that patients may be very unsettled or frightened due to previous online searches and fear that they may have a serious illness	82% (31) yes (often or at least occasionally),18% (7) no
Experienced challenges in caring for cyberchondriacal patients	74% (28) yes, 26% (10) no
Extensive research on the Internet that can ultimately trigger or exacerbate a cyberchondria	71% (27) yes, 29% (11) no
Cyberchondria often goes along with damaging the physician’s authority	58% (22) yes, 42% (16) no
Patients with cyberchondria need extra counseling time	92% (35) yes, 8% (3) no
Patients with cyberchondria call for further diagnostics	79% (30) yes, 21% (8) no
Patients with cyberchondria need psychologic assistance	58% (22) yes, 29% (11) no, 13% (5) uncertain
Already experienced the termination of a patient-to-doctor basis due to the patient’s extensive online research	21% (8) yes, 79% (30) no
Estimated cause of cyberchondria	31% only personality predispositions; 59% progressive development without having hypochondriacal tendencies in the first place

### Strategies in dealing with cyberchondria patients

The general practitioners were asked what kind of procedures they consider appropriate and promising in order to help patients who are unsettled or frightened by excessive investigations on the Internet or to prevent the onset of cyberchondria. Almost all interviewees mentioned more than one course of action they usually follow (commonly on the condition that significantly more time is given to the patient). Table 3 contains the categorized results.

**Table 3:** Procedures and strategies for dealing with Internet-related health anxieties/cyberchondria

Perceived cause of (emerging) cyberchondria	Implication from the GP’s standpoint	Specific approach	Focus	Percentage share in the sample (*N* = 38)
Disorientation and uncertainty due to the lack of a serious, competent medical authority guiding the patient and giving trustworthy information	The doctor should eliminate other sources of information that (potentially) conflict with his/her advice; the patient should be directed to the physician as a “decision-making and instructing authority” (I-23, male)	Actively discourage the patient from (further) health-related Internet investigations (for example, in case of uncertainty the GP can be contacted at short notice in order to prevent further rampant online searches and to strengthen the doctor–patient relationship)	Stabilization	21% (8)
Patients have a lack of understandable, transparent explanation as well as an insufficient overview of their treatment situation (for example, diseases, diagnoses, therapies, etc.)	The doctor can prevent a growing feeling of uncertainty and edginess in the patient by focusing on providing detailed information and clarifying questions	Detailed explanation of the diagnosis and therapy to prevent a patient from having an expansive or aimless internet research (provided that considerably more consultation time is granted)	Prevention/stabilization	63% (24)
Patients have a lack of serious, correct sources of information and advice; they are misinformed and misguided by the results of their health-related research	Physicians can correct patients’ false assumptions by providing the patient with alternative sources of information that are reputable and reliable; the patients’ aim to do independent research can be prevented	a) The patients are given recommendations to reputable health information sources on the Internet by the doctor (for example, certain health portals)	Prevention/stabilization	32% (12)
		b) The doctor refers to other information materials or hands them over (such as medical brochures, journal articles)		11% (4)
Patients wish to be taken seriously in their online research by the doctor and want their findings to be taken into account; if this is not the case, the patient turns (even more) to online research and becomes independent	In order to strengthen the doctor–patient relationship, the doctor should actively ask for and react to the patient’s Internet searches. This gives the patient appreciation and his/her research can ideally be used for further care	a) Joint examination and discussion of the health-related online information and health websites sought by the patient	Prevention/stabilization	26% (10)
		b) The doctor checks the information sought by the patient and tries to classify, correct, and clarify		13% (5)
In the early stages of cyberchondria, patients are often on their own and, in the course of their Internet searches, become nervous and panic-stricken, without the doctor being aware of it. If the tipping point is exceeded and health fears have become entrenched, it is often difficult to manage cyberchondria	Early detection of patients who do intensive health searches on the Internet with the doctor responding, if necessary, in order to prevent cyberchondria	a) Extension of the common medical history questionnaire in order to record the frequency of Internet research on health and illness on the part of the patient	Prevention	11% (4)
		b) For certain groups of patients, the doctor asks in principle whether previous health-related searches have been conducted on the Internet		18% (7)
		c) Fundamental thematization of chances and risks of online research during the consultation		11% (4)
Cyberchondria arises in a complex process of development and dynamization in which various psychosocial factors, personality traits, and experiences interact with each other	The doctor can only respond effectively to Internet-related health anxieties if he/she has the necessary background knowledge to influence affected patients competently; this is all the more important given the lack of psychotherapeutic care capacities	Doctors complete further psychosocial training (such as psychotherapy, psychoanalysis, social medicine)	Prevention/stabilization	11% (4)

*GP *general practitioner

The analysis revealed that the different approaches to stabilizing (potential) cyberchondriacal patients correspond to different perspectives on this category of patients as well as on the (presumed) causes of cyberchondria.

Some respondents believe that they need to emphasize their authority as general practitioner in order to help patients in the medium/long term. For this purpose, the patient is fundamentally advised to stop any search on the Internet. In this way, the confusion of patients with health anxieties is to be counteracted and the advisory work for the doctor to be facilitated.

However, this position only reflects a minority of the sample. Other physicians follow a more participative approach by offering detailed explanations during the doctor–patient interview and relying on communication as transparent as possible. In this context, the doctor not only inquires about the patient’s preliminary search, but also, if necessary, a joint examination and discussion of the searched information takes place or alternative reputable sources of information are recommended.

You know, I cannot stop my patients from looking things up on the Internet anyway. I cannot control that. Therefore, I have to make sure that something changes in the mind of the patient, even if he/she searches further. In my opinion, this is best when you signal to the patient that you listen carefully and take his/her concerns seriously. Involving the patient’s search into the conversation is always a good idea. From there it is much easier to earn the trust that is necessary (I-30, female).

A third group of interviewees focuses more on the preventive dimension. These physicians have set themselves the aim of becoming aware early of patients who are intensively searching on the Internet in order to have an early warning system for a potentially developing cyberchondria. As a matter of principle, certain patient “types” (e.g., patients with chronic illness, serious illness experience, or mental stress) are asked whether they have done health research before the doctor’s visit and sensitize them to possible risks of such behavior.

Last but not least, there are several interviewees who try to provide patients with psychosocial support through further training in the psychosocial field. These general practitioners show a strong sensitivity for the complexity of cyberchondria and think of it as not only resulting from personality traits.

I think there are certain techniques that can be learned to reassure patients and by which we can be as professional as possible. Occasionally, we make mistakes that we are not aware of—we send out the wrong ‘signals,’ which could then further unsettle or confuse these already unstable patients. By becoming aware of our mistakes through psychosocial trainings, we can avoid them (I-37, female).

### Dealing with extreme cases and optimization approaches

From the point of view of a majority of those interviewed, referencing of cyberchondriacal patients to mental health services is often not a viable option in the reality of care, as adequate care facilities are booked out over long periods of time or are not available in rural areas. As a result, general practitioners are often forced to find their own strategies to counteract acute health anxieties without having the professional qualifications and time they need. Some of the interviewees express clear displeasure that GPs are left alone in the care of mental stress and disorders.

Although it is legitimate to ask GPs for training to improve psychosocial care, additional care structures need to be created to help family physicians in dealing with phenomena such as cyberchondria (I-35, female).

The interviews showed that some general practitioners have started to think about improving primary care with regard to cyberchondria patients. In particular, the early prevention of Internet-related health anxieties is given great attention. One of the interviewees expresses this as follows:

It is completely unrealistic that we address all patients who have excessive Internet consumption. No, what we need is a tool or early-warning system which allows us to filter out exactly those patients who develop truly dysfunctional, pathological Internet use and are at risk of serious mental illness. It must be considered how we can achieve this. Everyone has a lot of catching up to do here. It will only succeed in a combination of training, education, and probably more mental health services […] and by better networking with them (I-19 male).

The development of a structured training for primary care physicians, which especially facilitates the identification of cyberchondria patients, is encouraged. Here, the practice staff can be effectively involved paying attention to warning signals. In addition, several interviewees would like to be better informed about serious, evidence-based Internet health portals that they can unhesitatingly recommend to patients.

## Discussion

### Main findings and comparison with prior work

So far, there are hardly any studies on how primary care providers perceive the cyberchondria phenomenon, what experiences they have made, and what approaches they pursue. In the course of analyzing the interviews, it has become clear that general practitioners see an increasing problem in the fact that some of their patients experience health anxieties due to intensive online investigations. The majority of interviewees perceive negative consequences for the psychological stability and wellbeing of patients, decreasing trust, and false expectations towards the doctor, as well as serious compliance problems resulting from excessive online searches that have caused a lot of confusion. One in five physicians had experienced the termination of one or more doctor–patient relationships because the patient’s online information behavior led to strong nervousness and panic states, which made further treatment impossible. In this respect, the results are consistent with the quantitative preliminary study [43] and with a discomfort noted in other studies which physicians have towards patients doing extensive online research [16, 30, 36, 39–42].

At the same time, the results show that general practitioners have begun to adjust to the phenomenon of cyberchondria and to develop strategies for dealing with that new challenge in everyday practice. In order to calm and stabilize insecure or frightened patients or to prevent the development of anxiety and panic states in the future, some of the physicians have got into the habit of asking certain patient groups for their online searches as a precautionary measure. The information that patients may have gathered will then be considered in the further doctor–patient interview.

Primarily, the general practitioners interviewed rely on preventing Internet-related health anxieties among their patients by taking sufficient time to explain a diagnosis, a therapy, or by providing background information on a clinical picture. In addition, some GPs recommend certain health portals they consider to be reputable so that patients can follow-up the doctor’s visit or conduct further searching. Some physicians are willing to grapple with the online information sought by the patient and to discuss the results. Last but not least, there are doctors who have undergone psychotherapeutic training in order to be better able to respond to cyberchondriacal patients.

Ahluwalia et al. have already emphasized the importance of the psychosocial professionalization of primary care physicians for successful treatment of patients suffering from anxiety disorder. This qualitative study shows that general practitioners can learn to be able to act successfully upon cyberchondriacal patients by using cognitive and behavioral techniques to respond appropriately to patients, buying time in a consultation and distancing themselves from their emotional response. Furthermore, GPs can learn “using the internet as an ally, by directing patients to particular websites” [42].

The good practice examples identified in the course of the analysis (see Table 2) are useful for making GPs aware of the possibilities they have to act on patients affected by health fears.

### Strengths and limitations

Due to the qualitative approach, the limited number of cases, and the regional recruitment in the federal state of Rhineland-Palatinate, Germany, the study must be considered as non-representative. It cannot be ruled out that greater numbers of general practitioners took part who already had an interest in the topic. The study was conducted in the German general practitioner care context, with its own specifics.

In the course of the study, GPs were asked how they perceive their online searching or cyberchondria patients and how they deal with corresponding health anxieties. Correctly, this perspective should now be contrasted with a patient survey to get a better understanding of the perspective of patients already affected by Internet-related health concerns [23].

For further investigations in the field of cyberchondria, Eichenberg suggests that these should aim to better capture possible factors of dysfunctional handling of health-related information and not to problematize potential effects of the Internet globally [27, 28]. A similar view has also been voiced by some of the general practitioners interviewed in this study. These interviewees want a toolkit for primary care physicians in order to filter out exactly those patients who tend to develop a dysfunctional, pathological use of the Internet in a targeted and early manner. It would also be worthwhile exploring in clinical trials which communication strategies used by doctors succeed in stabilizing patients with Internet-related health concerns.

## Conclusion

Today, physicians need to be prepared for patients who obtain information on health and illness issues online before and after their visit to the doctor. This does not remain without consequences for the doctor–patient relationship but can have a direct or indirect influence on the health-related behavior of patients, their appearance during a doctor’s visit, or compliance. Therefore, it can happen that extensive health-related online searching causes health anxieties in patients which solidify in a long-term process.

The fact that the Internet-informed patient changes the traditional doctor–patient relationship is evident and is also reflected in the results of this study. In order to be able to influence Internet-related health anxieties in general practitioner care, it seems advisable to actively discuss the online information search in the patient interview and, especially when dealing with hypochondriacal patients, to address its chances and risks.

It seems very important that doctors pay increased attention to anxious patients so that they do not feel compelled to search for answers and solutions on the Internet on their own, thereby damaging the doctor–patient relationship. Various doctor–patient communication literature emphasizes how important it is for the physician to give appreciation to the patient in conversation (e.g., [45, 46]). By considering the patient’s previous knowledge and giving him/her the feeling of actively contributing to his/her examination, diagnosis, or therapy, it is possible to include additional, more complete or pertinent information, and, at the same time, to strengthen the doctor–patient relationship by opening lines of communication between doctors and patients [45, 47]. In this way, a trustful relationship can be built even with difficult patients. Against the background of the interview results, such a communication strategy appears particularly suitable for the clinical picture of cyberchondria. On the one hand the patient can be given the feeling of taking his/her online searches seriously, on the other hand the doctor–patient relationship can be prevented from being damaged by an autonomous patient online search behavior, because there is now a stronger reconnection to the doctor’s advice.

Moreover, it would be worthwhile to extend the medical history by the dimension of online information search in order to have an early view of the emergence of possible health fears. It should also be taken into account that hypochondriacal patients or patients who are unsettled due to contradictory or incorrect information on the Internet may need more consultation time. Here, the doctor’s interview should follow the basic recommendations for dealing with patients with somatoform disorders [15, 22].

Last but not least, it makes sense to increase the awareness of serious, evidence-based online information on health issues—not only among patients but also among doctors [40, 48, 49]. This kind of overview would make it easier for primary care physicians to refer patients to websites that pose no risk of misinformation or confusion, so that there is a reliable source of information for patients who want to undertake further searching on the Internet. Professional societies and professional associations could help physicians in private practice to gain a better overview of health sites.

If Internet-related health anxieties have progressed too far and the general practitioner is not able to stabilize such patients, it will be important that there is a sufficient range of low-threshold mental health services that doctors can easily and quickly refer to. Considering the lack of available psychotherapeutic care capacities, compact online therapy services specially for cyberchondria patients could be valuable.

## Caption Electronic Supplementary Material

Interview guidelines
