# Supplementary material for: General practitioners’ challenges and strategies in dealing with Internet-related health anxieties—results of a qualitative study among primary care physicians in Germany
Source: Wien Med Wochenschr. 2020 Aug 7;170(13):329–39. doi: 10.1007/s10354-020-00777-8 (PMC7518985; doi:10.1007/s10354-020-00777-8)
Supplement: Supplementary file 1 — Interview guidelines [file 10354_2020_777_MOESM1_ESM.docx]

**Interview Guidelines**

**1.** The Internet offers patients many opportunities to find out about health and illness issues, ‘googling’ their own complaints, or exchanging complaints with other users.

a) Do you consider that to be of more positive or negative consequences for the doctor-patient relationship? Why?

b) How do you assess the consequences for the health behavior of patients?

c) Do you have the impression that patients who are intensively searching on the Internet about health and illness issues are better informed and do know more about symptoms, diagnoses or therapies? Why (not)?

**2.** Please describe what experiences you have had with patients who are searching on the Internet for information on health and illness or even possible diagnoses and therapies. What did you notice about these patients?

**3.** The relationship between doctor and patient may change as patients regularly get information about health and disease issues from the Internet. What did you experience in this regard?

**4.** What is the proportion of your patients who occasionally or more frequently confront you with health information from the Internet?

a) In which contexts, according to your observation or assessment, do patients primarily search for health-related information on the Internet (topics, preparation or follow-up of doctor’s visit…)?

**5.** How would you characterize patients who have a strong affinity to online health research? What is 'typical' for these patients?

**6.** Again and again, one hears and reads about patients who have been greatly unsettled or frightened by previous internet searches and, as a result, fear that they have a bad illness, although from a medical point of view there is no indication for this. In this context the term 'cyberchondria' is being used.

a) When you think of your patients: How often do you notice such unfounded fears of having a serious illness resulting from excessive Internet searches?

b) Would you say that patients who are scared or panicked due to excessive Internet searches are becoming an increasing problem in primary care?

**7.** In what way do you experience the care and handling of these patients as a challenge? What exactly do you find challenging? (Behavior during the consultation, compliance, time-intensive care, demand for further diagnostics…)

**8.** Has it ever happened that the care of a patient was canceled by the patient or by you because the patient was negatively influenced by his/her health searches on the Internet so that further care and a working doctor-patient relationship was no longer possible on that basis?

**9.** If you try to characterize patients who are affected by cyberchondria: From your experience, what characteristics are ‘typical’ of these patients?

a) How do you see this: Are only very specific individuals vulnerable to cyberchondria because they have, for example, certain hypochondriac personality traits, or can, in principle, anyone be affected by cyberchondria?

**10.** In what way do you use information from the Internet to assist you in your medical work (for example, referral to specific information services, such as certain health portals, that allow patients to follow up on the doctor's visit or to get more information)?

a) How often do you recommend certain websites to your patients where they can look up information about health and illness or even diagnoses and therapies?

**11.** In what way do you specifically ask certain patient types or groups whether they have conducted health-related research on the Internet?

a) To what extent do you take the patient‘s pre-searches into account during your consultation hours?

**12.** The general practitioner may take certain strategies or measures to calm or stabilize patients whose mental health status has been adversely affected by excessive Internet research on health and illness issues.

a) In this context, do you have any specific procedures or strategies to reassure patients who are nervous, desperate or in panic by their online searches or to prevent cyberchondria from arising?

b) Which approach do you consider to be promising and practicable? Where would a GP have to start in order to prevent or counteract cyberchondria?

c) Which needs, wishes or expectations on the part of the patients should one meet?

**13.** To what extent should GPs signal more strongly that they consider themselves as point of contact for patients who seek information on health and illness on the Internet (for example, by discussing the information with the patient or commenting it or providing research recommendations)?

a) What requirements must be met for this?
